# Development of the global climate crisis risk perception scale (GCCRP-S): A validity and reliability study

**DOI:** 10.1371/journal.pone.0321836

**Published:** 2026-09-09

**Authors:** Nalan Ozen, Murat Topbas, Nazim Ercument Beyhun, Erol Nezih Orhon, Ahmet Altin, Sevil Turhan

**Affiliations:** 1 Karadeniz Technical University, Faculty of Medicine Department of Public Health, Trabzon, Turkey; 2 Anadolu University Faculty of Communication Sciences, Department of Cinema and Television, Eskisehir, Turkey; 3 Zonguldak Bulent Ecevit University, Faculty of Engineering, Department of Environmental Engineering, Zonguldak, Turkey; Instituto Federal de Educacao Ciencia e Tecnologia Goiano - Campus Urutai, BRAZIL

## Abstract

Despite the increasing scientific data on the causes and effects of the global climate crisis, it is seen that the perceptions, approaches, practices, views and opinions of both governments, some organizations and institutions, and individuals and societies are divided. In order to clearly reveal this situation, this study develops a global climate crisis risk perception scale. A methodological research was carried out in 8 stages: determining the boundaries of the concept/structure; creating of the item pool; submitting the item pool to expert opinion; pilot study; preparation of the data collection tool; main application with large group; test-retest application; statistical analysis and finalization of GCCRP-S. The draft scale was applied at face-to-face interviews with 749 participants. Content, Face, Construct and Concurrent Validity were evaluated to determine validity. Internal consistency and stability tests were used to determine reliability. A five-factor structure emerged that explained 69.40% of the variance. Goodness of fit indices were within the values reported in the literature. The Cronbach’s alpha coefficient, which shows the internal consistency, was 0.957. The intraclass correlation coefficient, indicating the stability, was 0.940. The GCCRP-S provides an initial multidimensional framework for examining global climate crisis risk perception in adults. Following validation in independent and more diverse populations, it may support future research and provide contextual evidence for climate-risk communication and policy discussions.

## 1. Introduction

The global climate crisis (GCC) is one of the most important environmental public health problems of the 21st century [1]. Its effects are being felt with increasing intensity across the world [2,3]. The Lancet Countdown Report emphasizes that the current situation, although having a disproportionate and unequal impact on different societies, is felt on every continent, and is gradually worsening [4]. As stated below, the global climate crisis has direct or indirect negative effects in many areas, both at the global and regional levels.

• Change in the climatic characteristics of the seasons: Extreme heat and cold weather waves, irregularities in the amount and regime of precipitation [5]
• Effects on nature and natural events: Increasing severity and frequency of floods, drought, desertification, storms, typhoons, hurricanes, tornadoes, and forest fires [6]
• Effects on ecosystems and biodiversity: Disruption of the functioning of terrestrial, marine, and polar ecosystems, loss of biodiversity [7–11]
• Effects on agriculture and food security: Reduction of agricultural lands, negative impacts on agricultural diversity and productivity [12]
• Effects on livestock: Decreases in and extinction of animal species, reduced animal food production [13]
• Effects on water resources and water security: Decreases in clean and safe water resources, decrease in water quality [14]
• Effects on fisheries: Reductions in the numbers of fish and fish species living in fresh and salt waters [15]
• Effects on the economy: Adverse effects on the food sector, tourism activities, energy sector [16–18]
• Effects on well-being of living things: Emergence of new microorganisms or revival of previously known microorganisms, epidemics of infectious diseases, poor air quality, food crisis, water crisis [19–22]
• Social effects: Migration, famine, security issue, disputes [23]
• Effects on human health: Increased morbidity and mortality [24–26]

Intensive efforts are required to prevent further worsening of the GCC, to ensure protection from the effects experienced, and to achieve permanent and sustainable results. Although the primary measures to be adopted to combat the crisis involve action on the part of governments and the enshrining of regulations with an institutional and sector based approach, not only governments and institutions, but also all actors and individuals in society must act together, take responsibility, and behave appropriately [1,27,28]. Despite the increasing scientific data regarding the causes, effects, and potential risks of the GCC, the perceptions, approaches, practices, views, and opinions of governments, various organizations and institutions, and societies and the individuals that comprise them are all divided. There is also a discord between society and administrators [29–32]. In short, there is a chaotic situation on a global level. It is essential to determine the global climate crisis risk perceptions of both individuals and society to clearly identify this chaotic situation.

Risk perception is defined as an individual’s subjective beliefs and value judgments concerning the possibility of and potential harm that will result from a risk [33]. If individuals believe that a situation will cause them serious harm, they think that the risk will diminish when they take action to reduce it. They therefore control their risky behaviors, exhibit protective behaviors, and adapt to the measures taken. They also encourage other people around them to adopt similar protective behaviors [34,35]. The perception of the risk posed by a given situation thus plays an important role in determining and motivating individuals’ behavior in the face of it [36].

Risk perception is evaluated through two components. The first is the cognitive component, which refers to individuals’ cognitive judgments about the likelihood and magnitude of risks. Second is the emotional component, which focuses on individuals’ emotional judgments and attitudes such as fear, anxiety and anger. Considering these two components, it is seen in the literature that risk perception is examined in different sub-dimensions: perceived probability, perceived gravity, perceived anxiety, perceived insecurity, perceived sensitivity, perceived benefits, and perceived obstacles [37–43].

A review of the literature shows that field studies on the global climate crisis, or formerly referred to as climate change, have focused on awareness along with risk perception. In fact, in the national literature, a scale was developed to assess climate change awareness, and subsequently, the extent to which climate change awareness varied according to certain sociodemographic variables was examined. These studies, which explore the concept of awareness defined as the timely perception and understanding of the global climate crisis and its impacts are important for raising public consciousness so that societies can adapt socially, economically, and environmentally to the effects of the global climate crisis and minimize its potential adverse consequences. However, for people to break away from established patterns, take action, and be willing and determined to adopt protective measures against adversities, it is necessary that they perceive the situation they are aware of as a risk. The observed outcomes of global climate change, as well as its likely future impacts, may affect individuals’ mental health and lead to reactions such as fear, stress, trauma, depression, anxiety, and worry. Similar to the emphasis on awareness, in the literature, anxiety and worry among these psychological reactions—have become the focus of research on the psychological effects of climate change, leading researchers to develop measurement tools to assess climate change anxiety and climate change worry. While these studies are important in revealing the relationship between the climate crisis and mental health, they do not provide clear information on behavioral engagement or adaptation. Moreover, risk perception is often limited to expressing only the emotional dimension of risk, and remains insufficient in assessing the cognitive dimension of risk namely, individuals’ sensitivity and the severity of the risk they perceive.

By determining the risk perceptions of individuals regarding the global climate crisis, firstly, their awareness of the possibility of the crisis harming individuals, their families, children, grandchildren, future generations, and society will be evaluated. In addition, individuals’ concerns about the damage that may occur due to the crisis and the severity of this damage will also be evaluated. It is envisaged that the determination of the global climate crisis risk perception can guide the adoption and support of existing international policies to combat the crisis, and the development and implementation of national policies. In the studies conducted, it is stated that adopting protective action recommendations by individuals and society may be related to the global climate crisis risk perception. It is emphasized that global climate crisis risk perception can be an effective mediating factor and a fundamental component of behavioral change between national or international policies and the behaviors of individuals and society [36].

Determination of global climate crisis risk perception may also be relevant to existing local, regional, and national policies and practices. As the climate crisis is closely associated with multiple dimensions of vulnerability, assessing risk perception may provide an empirical basis for identifying patterns of perceived vulnerability and developing vulnerability profiles across population groups. Such profiles may help local, regional, and national governments, non-governmental organizations, policymakers, and environmental groups tailor communication, preparedness, adaptation, and risk-reduction interventions to the needs of groups with different vulnerability profiles. Previous studies have examined several of these components, although generally as separate constructs or within explanatory models. Van der Linden’s Climate Change Risk Perception Model integrated cognitive and experiential/affective factors to explain personal and societal climate risk judgments [44]. Emotional responses to climate change have also been operationalized through measures such as the Climate Change Worry Scale [45]. In addition, perceived health risks have been examined as a construct related to, but distinct from, general perceptions of harm [46], while response efficacy and psychological adaptation—constructs conceptually related to perceptions of crisis management capacity—have been evaluated in models linking climate risk perception to pro-environmental behavior [47]. However, these approaches have generally treated these components as separate constructs, predictors, or outcomes rather than as dimensions of a single psychometrically validated instrument. To our knowledge, no comprehensive and multidimensional scale has integrated cognitive appraisal, emotional response, perceived health risks, and perceptions of crisis management capacity within a single framework for assessing global climate crisis risk perception. Therefore, this study aimed to develop a Global Climate Crisis Risk Perception Scale (GCCRP-S) to determine risk perception toward the GCC in adults. This study aimed to develop a Global Climate Crisis Risk Perception Scale (GCCRP-S) to determine risk perception toward the GCC in adults.

## 2. Materials and methods

This research was designed as a methodological study. The scale development process was discussed comprehensively in 8 stages [48–52]. The stages involved are described below:

### 2.1. Determining the characteristics and boundaries of the concept/structure to be measured

Due to the different approaches to the manner in the current situation is expressed, we examined studies at both national and international levels and related to three different terms: “global warming”, “climate change” and “global climate crisis”. We also investigated whether any measurement tools had previously been developed or adapted for this subject. Examination revealed that no objective scale was available for determining the risk perception in the context the GCC, and it was therefore not possible to clearly determine whether individuals perceive the crisis as a risk. We determined the conceptual structure, features, and boundaries of the concept/structure to be measured, to establish the conceptual framework, and to determine the original aspects of the model that would emerge [50]. A new conceptual framework model has been created that is predicted to explain a significant amount of variance in global climate crisis risk perception and help integrate different theoretical perspectives.

### 2.2. Creating the item pool

Taking into account the determined conceptual framework model, information suitable for individuals and society was brought together, and draft items were prepared to measure/determine the risk perception towards the global climate crisis. The consensus on the number of items is to prepare as many items as possible [50,53,54]. An item pool of 64 draft items was created to evaluate the global climate crisis risk perception. An even-numbered, six-point Likert-type response format was used to assess the level of agreement with each item. The format comprised three disagreement and three agreement categories and did not include a neutral midpoint. All response categories were verbally labeled and scored in ascending order as follows: 1 = Completely Disagree, 2 = Disagree, 3 = Partially Disagree, 4 = Partially Agree, 5 = Agree, and 6 = Completely Agree [55]. Both positive and negative items were included to avoid bias [50]. It was envisaged that the prepared draft items would be divided into five factors: “Perceived probability”, “Perceived gravity”, “Perceived sensitivity”, “Perceived way of management” and “Situations that prevent risk perception”.

### 2.3. Submitting the item pool to expert review

At this stage the content validity was assessed. Each item and the extent to which the scale as a whole serve the purpose is referred to as content validity [56–58]. There are various methods for determining the content validity. We preferred the most widely used Lawshe technique [59–61]. In Lawshe Technique, each expert was asked to assign a score of 1–3 to each item depending on its suitability for measuring the desired attribute. A score of 3 meant the item was “suitable”, a score of 2 meant it was “suitable but needs to be more appropriate”, and a score of 1 meant it was “unsuitable”.

In order to determine the content validity of the items to be included in the scale, the qualitative data obtained in line with expert opinions are converted into quantitative data by calculating the Content Validity Ratio (CVR), Critical Content Validity Ratio (CVRcritical) and Content Validity Index (CVI). Taking these values into consideration, the final items that will remain in the candidate scale are determined [62].

Experts were purposively selected based on their academic and professional experience in public health, environmental sciences, risk perception, climate change, and scale development. The expert panel was designed to ensure conceptual diversity rather than disciplinary symmetry. In cases of divergent expert opinions, items were re-evaluated by the research team through consensus discussions, with revisions guided by theoretical relevance and clarity rather than CVR values alone.

The CVR is calculated according to the formula “(Ns/(N/2)) – 1” (N: number of experts; Ns: number of experts describing the item as suitable) [62]. An item with a zero or negative CVR value is considered to have no content validity and is eliminated from the scale. The CVRcritical value is determined according to **Table 1** in the literature and varies depending on the number of experts [59–61]. The CVI is determined by calculating the mean of the CVR values of the remaining items. If the calculated CVI value is greater than the CVRcritical value, this indicates that the content validity of the remaining draft items is statistically significant [62].

**Table 1:** Critical content validity ratio (CVRcritical) according to the Lawshe Technique.

Number of experts	Minimum value	Number of experts	Minimum value
5	0.999	23	0.391
6	0.999	24	0.417
7	0.999	25	0.440
8	0.750	26	0.385
9	0.778	27	0.407
10	0.800	28	0.357
11	0.636	29	0.379
12	0.667	30	0.333
13	0.538	31	0.355
14	0.571	32	0.375
15	0.600	33	0.333
16	0.500	34	0.353
17	0.529	35	0.314
18	0.444	36	0.333
19	0.474	37	0.297
20	0.500	38	0.316
21	0.429	39	0.333
22	**0.455**	40	0.300

### 2.4. Pilot study

This stage involves the determination of face validity. Face validity indicates that a scale indeed seems to measure what it is intended to measure. The pilot study includes a specification of what the draft scale appears to measure and the evaluation of each item in terms of issues such as appearance, readability, intelligibility, and difficulty/ease of answering [54,63–65]. The scale’s Cronbach’s alpha (α) coefficient, corrected item-total correlations, Cronbach’s α coefficients of the draft scale if the items deleted are calculated by analyzing the data obtained from the pilot study. The Cronbach’s α is used to explain the homogeneous structure. The items in the scale with a high Cronbach’s α coefficient consist of items that are consistent with each other and that measure the same feature [58]. The adequate threshold value for the Cronbach’s α coefficient is ≥ 0.70 [66]. Corrected item-total correlation refers to the correlations between the score obtained from each item in the scale and the total scale score. If the corrected item-total correlation value is negative, zero, or close to zero, this means that the item contributes little to the measurement of the construct to be measured and is insufficient to measure it that construct. These items should not therefore be included in the scale. The common view in the literature is that item-total correlation should be ≥ 0.30 [67–70].

In the literature, it is stated that the pilot study should be conducted on individuals who are similar to the research sample but will not participate in the main study, in order to ensure impartiality. However, there is no consensus on the number of participants required for a pilot study, and different studies suggest different sample sizes [54–64]. In this research, the pilot study was conducted with 50 individuals who were selected through convenience sampling, in line with the inclusion and exclusion criteria, and who were similar to the larger sample group but would not take part in the field application of the study. Data for the pilot study were collected through face-to-face interviews.

Corrected item-total correlations, Cronbach’s α coefficients if the items deleted, and the scale’s Cronbach’s α coefficient were calculated.

### 2.5. Preparation of the data collection tool to be used in the main application, including the draft scale

Adjustments were made to the draft items in line with the expert opinions and suggestions, the suggestions received from the participants through the pilot study, and the results of the item analysis. The finalized data collection form consists of five parts.

In the first part, participants were asked about their sociodemographic characteristics (sex, age, marital status, having children, education, profession). In the second part, the GCCRP-S planned to be developed was included and participants were asked to answer 45 items. The 45-item draft GCCRP-S used the six-point Likert-type response format described above.

In the third part, the Attitude Scale Towards Environmental Problems is included. The scale was developed in 2009 to determine individuals’ attitudes towards environmental problems. The scale consists of 11 items collected under a single factor. The scale’s Cronbach α was calculated at 0.80. Scores obtained from the scale are between 11–55. High scores indicate a positive attitude toward environmental problems [71]. The fourth section contained the Climate Change Awareness Scale developed in 2022 to measure individuals’ awareness of climate change. This consists of 52 items collected under five factors. The scale’s Cronbach α was calculated at 0.92. All the sub-dimensions of the scale can be used separately. In this study, “Climate Change Awareness” sub-dimension was employed. Scores obtained from the scale are between 9–45. High scores indicate high climate change awareness [72]. The fifth section includes the Climate Change Concern Scale, which was developed in 2021 to measure individuals’ concerns about climate change [45] and adapted to Turkish in the same year. It consists of 10 items collected under two factors. The scale’s Cronbach α coefficient was calculated at 0.91. Scores obtained from the scale are between 10–50. High scores indicate high concern over climate change [73].

### 2.6. Main application with a large sample group

Different approaches can be employed for determining the sample size in scale development studies [74]. In this study, the sample size was calculated as 675, with 15 individuals per item. However, we planned to enroll a minimum of 700. Stratification was performed based on gender and age distribution. The draft scale was applied through face-to-face interviews with participants who were reached by the convenience sampling method.

• Exclusion criteria: Being under the age of 18 or over 60; being mentally inactive, uncooperative, or uncommunicative; illiteracy; not being able to speak Turkish; being unwilling to participate in research. Lack of formal education and advanced age may affect the understanding and interpretation of questions containing current and technical information. Therefore, illiterate individuals and elderly people were not included in the study.
• Inclusion criteria: Being between the ages of 18 and 60; being mentally active, cooperative, and communicative; preferably being at least a primary school graduate; being able to speak Turkish; voluntary participation in the research

This study was conducted in the Ortahisar district of Trabzon, located in the Eastern Black Sea Region of Turkey. In the first stage of the study, six different neighborhoods with no adjacency to each other were identified. Subsequently, places such as cafés/coffee houses, restaurants, grocery stores/markets, patisseries, workplaces, mosques, and parks located in these neighborhoods were visited, and individuals present in these venues were provided with the necessary information about the research. Written consent was obtained from those who agreed to participate. After obtaining written consent, the data collection form was administered face-to-face. A relationship of trust was established with the participants, and thanks to this trust, a sufficient number of participants were reached in a short time, and the data were collected completely and accurately.

Prior to the research, approval was obtained from Karadeniz Technical University Scientific Research Ethics Committee (decision dated 01.12.2022 and numbered 24237859−666). Before starting data collection, each participant was given detailed information about the research verbally. In addition, detailed information about the research was given in the introductory part of the data collection form and the participants were given the necessary time to read this part before data collection. Therefore, the participants were informed both verbally and in writing, and as a result, the process continued with those who agreed to participate in the research. Signatures were obtained from the participants indicating that they volunteered and agreed to participate in the study. For the retest to be conducted three weeks after the first data collection, the name, surname, address and telephone number information was obtained from 58 participants who volunteered for the retest to be conducted three weeks after the first data collection. Participants provided written consent during the enrollment process. Data were collected between May 29 and July 10, 2023.

### 2.7. Test-retest application of the draft scale to determine its stability

The ability of a measurement tool to provide consistent results from application to application is measured by test-retest reliability [75]. The relationship between the participants’ responses to the first test and the retest is evaluated with the Spearman rank correlation coefficient and the intraclass correlation coefficient (ICC). The correlation coefficient between the scores gives the reliability coefficient of the scale [76]. A statistically significant, positive, and high correlation is expected between the responses to the first test and the retest. In this study, 58 participants, whose contact information (name, surname, telephone number, and address) was obtained, were retested 3 weeks after the main application.

### 2.8. Statistical analysis of data, and finalization of the GCCRP-S

IBM SPSS (Statistical Package for the Social Sciences) version 26.0 was used for descriptive statistics for the research group, descriptive statistics for items, item analyses, exploratory factor analysis, reliability analyses (Cronbach’s α coefficient and ICC), and concurrent validity. IBM AMOS (Analysis of Moment Structures) version 28.0 software was used for confirmatory factor analysis. A p value of <0.05 was considered statistically significant.

#### 2.8.1. Validity analyses.

Validity expresses the degree to which a measuring instrument can accurately calculate the feature it aims to measure without confusing it with any other feature [57]. At this stage, the construct validity of the draft scale was determined. Construct validity describes how well the scale correlates with the structure being measured, and it indicates the scale items are compatible, complement one another, and form a whole collectively [58,77,78]. The construct validity of the scale was tested using exploratory factor analysis and confirmatory factor analysis.

**2.8.1.1. Exploratory factor analysis (EFA).** The suitability of the data for factor analysis was evaluated using the Kaiser–Meyer–Olkin (KMO) measure of sampling adequacy and Bartlett’s test of sphericity. The KMO statistic assesses sampling adequacy and the factorability of the correlation matrix, with values ≥0.50 considered acceptable. Bartlett’s test of sphericity examines whether the correlation matrix differs significantly from an identity matrix; a statistically significant result (p < 0.05) indicates that the correlations among the items are sufficient for factor analysis [58,79,80].The fact that the chi-square value obtained as a result of this test is statistically significant (sig. < 0.05) means that it is appropriate to conduct factor analysis with the correlations between the items and the available data [58,81,82]. Common factor variance values (communalities) show the variance explained by the factors in each item. In this study, items with a common factor variance value >0.40 were included in the scale [83]. Various methods can be used to reveal the factor pattern of a scale. In this study, the Principal Components Analysis was used. It is stated that no distribution assumption is required in this method. Additionally, it allows extracting the highest variance from the data set [84].

Various rotation methods must be used to reach the best model in EFA. The purpose of factor rotation is to maximize the loading of an item on a factor and to minimize the loading of the same item on other factors. There are oblique and orthogonal rotation methods in factor rotation. There are various criteria regarding which rotation method will be applied. According to the general approach, if there is a relationship between the factors, oblique/oblique rotation methods (Direct oblimin, Promax) are used, and if each of the factors is different from the others, orthogonal/orthogonal rotation methods (Varimax, Quartimax, Equamax) are used [84]. In this study, direct oblimin was employed among the oblique rotation methods.

Items with factor loading values ≥0.40 were included in the scale. When an item was loaded under more than one factor and the difference between factor loading values was < 0.10, that item was regarded as an overlapping item [85]. Care was taken to avoid problems such as multicollinearity and singularities. Multicollinearity is a strong relationship between two items (r > 0.90), while singularity means that the correlation value between the scale item pairs is equal to 1.00 [86]. In addition, items with very low correlation (r < 0.10) with other items were not included in the scale.

The number of factors to be retained was determined by jointly considering the scree plot, eigenvalues, cumulative variance explained, and the conceptual interpretability of the factor solution. The Kaiser criterion (eigenvalue ≥1) was used as one of several indicators rather than as a definitive decision rule [58,81,82]. During the EFA experiments, whenever violations of these criteria were encountered, the relevant item was examined and, if necessary, removed from the scale. Analyses were repeated to create various models. Ten EFA experiments were conducted until the model yielding the best results was reached. The resulting model consisted of 29 items and 5 factors. Each factor had a loading of at least 4 items.

In the scree plot, there are eigenvalue numbers on the horizontal axis and eigenvalue magnitudes on the vertical axis. Points with steep slopes are considered. The intervals between the points on the horizontal line drawn from the point where the graph transitions to horizontal slope are counted. Each interval between two points represents a factor [87].

**2.8.1.2. Confirmatory factor analysis (CFA).** Confirmatory factor analysis is used to verify the model formed by the factors obtained from EFA. CFA has two main purposes. The first is to test the relationships between factors and items, and the second is to test the adequacy of the items in explaining the factors and the theoretical structure in general. Goodness-of-fit values are used to evaluate the fit of the model in CFA. CMIN/df (Minimum Discrepancy Function by Degrees of Freedom divided), AGFI (Adjusted Goodness of Fit Index), CFI (Comparative Fit Index), NFI (Normed Fit Index) and RMSEA (Root Mean Square Error of Approximation) goodness-of-fit values were determined in this study [64,88,89].

**2.8.1.3. Concurrent validity.** Concurrent criterion validity shows the predictive power of the draft scale. To determine concurrent validity, scales developed to measure another construct thought to be similar or related to the construct to be measured, together with the draft scale, are applied to the participants under the same conditions [90]. A statistically significant, positive, and moderate correlation is expected between the scores obtained from the draft scale and others. The Attitude Scale Towards Environmental Problems, the Climate Change Awareness Scale, and the Climate Change Concern Scale were employed in this study. After applying the scales, scale scores were analyzed using Spearman correlation.

#### 2.8.2. Reliability Analyzes.

Reliability refers to the stability and consistency of the measuring instrument over time. A reliable scale should yield similar results between applications. In this study, we used internal consistency and stability to assess reliability [91]. To determine internal consistency, the following values are calculated: corrected item-total correlations, Cronbach’s α values when the items are deleted, and Cronbach’s α of the scale and its subdimensions. For stability checks, the retest occurred three weeks after the main application with the volunteer group. The Spearman rank correlation coefficient and the intraclass correlation coefficient (ICC) are used to assess the relationship between the participant’s responses to the initial test and retest. ICC values > 0.75 are regarded as perfect [92,93].

## 3. Results

### 3.1. Participants

Seven hundred forty-nine individuals participated in the research. The mean and standart deviation of participants’ ages was 37.2 ± 11.7 (min-max = 18–60), and 51.1% (n = 383) were women. In addition, 62.2% (n = 466) of the participants were married and 60.6% (n = 454) had children. In terms of education levels, 49.4% (n = 370) were university graduates, while 25.6% (n = 192) were in specialist professions.

### 3.2. Validity analysis results

#### 3.2.1. Content Validity.

The opinions of 22 experts were received. The CVRcritical was determined as 0.455 (Table 1). Expert opinions regarding the items, the CVR of each item, and the CVI of the draft scale are shown in Table 2. Two items (I3 and I5) with a CVR less than the CVRcritical value were excluded from the scale. Items that needed to be made more appropriate or removed entirely according to the experts’ opinions were additionally examined and 12 of the 62 items were accordingly deleted from the scale. The admitted items were given their final forms in the light of minor changes recommended by the experts. A draft scale form consisting of 50 items was thus established. The CVI (0.831) calculated with the remaining items exceeded the CVRcritical value (0.455).

**Table 2:** Expert opinions regarding the items andntent validity ratios.

	Expert scores					Expert scores			
Item	3	2	1	CVR	Item	3	2	1	CVR
I1	20	2	–	0.818	I33	20	2	–	0.818
I2	20	2	–	0.818	I34	21	1	–	0.909
I3	15	6	1	0.363*	I35	20	2	–	0.818
I4	18	2	2	0.636	I36	22	–	–	0.999
I5	14	5	3	0.272*	I37	20	1	1	0.818
I6	19	2	1	0.727	I38	19	3	–	0.727
I7	18	4	–	0.636	I39	22	–	–	0.999
I8	17	4	1	0.545	I40	22	–	–	0.999
I9	20	2	–	0.818	I41	21	1	–	0.909
I10	17	5	–	0.545	I42	21	1	–	0.909
I11	21	1	–	0.909	I43	21	1	–	0.909
I12	21	1	–	0.909	I44	20	2	–	0.818
I13	22	–	–	0.999	I45	19	3	–	0.727
I14	20	2	–	0.818	I46	21	1	–	0.909
I15	22	–	–	0.999	I47	22	–		0.999
I16	19	3	–	0.727	I48	20	1	1	0.818
I17	19	3	–	0.727	I49	21	1	–	0.909
I18	21	1	–	0.909	I50	21	1	–	0.909
I19	19	3	–	0.727	I51	21	1	–	0.909
I20	17	5	–	0.545	I52	20	1	1	0.818
I21	20	2	–	0.818	I53	19	3	–	0.727
I22	21	1	–	0.909	I54	18	2	2	0.636
I23	21	1	–	0.909	I55	19	3	–	0.727
I24	21	1	–	0.909	I56	21	1	–	0.909
I25	22	–	–	0.999	I57	22	–	–	0.999
I26	17	4	1	0.545	I58	21	1	–	0.909
I27	21	1	–	0.909	I59	22	–	–	0.999
I28	18	4	–	0.636	I60	20	2	–	0.818
I29	17	5	–	0.545	I61	17	5	–	0.545
I30	18	3	1	0.636	I62	22	–	–	0.999
I31	22	–	–	0.999	I63	21	1	–	0.909
I32	19	3	–	0.727	I64	19	3	–	0.727

Scale Content Validity Index: 0.831

*Items with a CVR value below the CVRcritical value.

Critical Content Validity Ratio (CVRcritical): 0.455.

Number of experts: 22.

CVR: Content Validity Ratio.

3 = suitable, 2 = somewhat suitable but needs to be made more appropriate, 1 = unsuitable.

#### 3.2.2. Face validity.

After the pilot study, item analyses (Corrected item-total correlations and Cronbach’s α coefficients) were performed. Since the item-total correlation of five items was < 0.30, these items were excluded from the draft scale. At this stage, in which face validity was ensured, five items were removed and two items were corrected in line with the participants’ suggestions. The main application thus continued with 45 items. As a result of the pilot study, Cronbach α coefficient of the draft scale was determined as 0.914.

At this stage, where face validity was ensured, the content of the remaining items was re-examined, and some of the initially proposed factors were revised: “Perceived way of management” was renamed as “Crisis management perception” and “Situations that prevent risk perception” was revised as “Perceived of importance”. However, these proposed factors were not adhered to during the EFA.

#### 3.2.3. Construct Validity.

**Fig 1:**
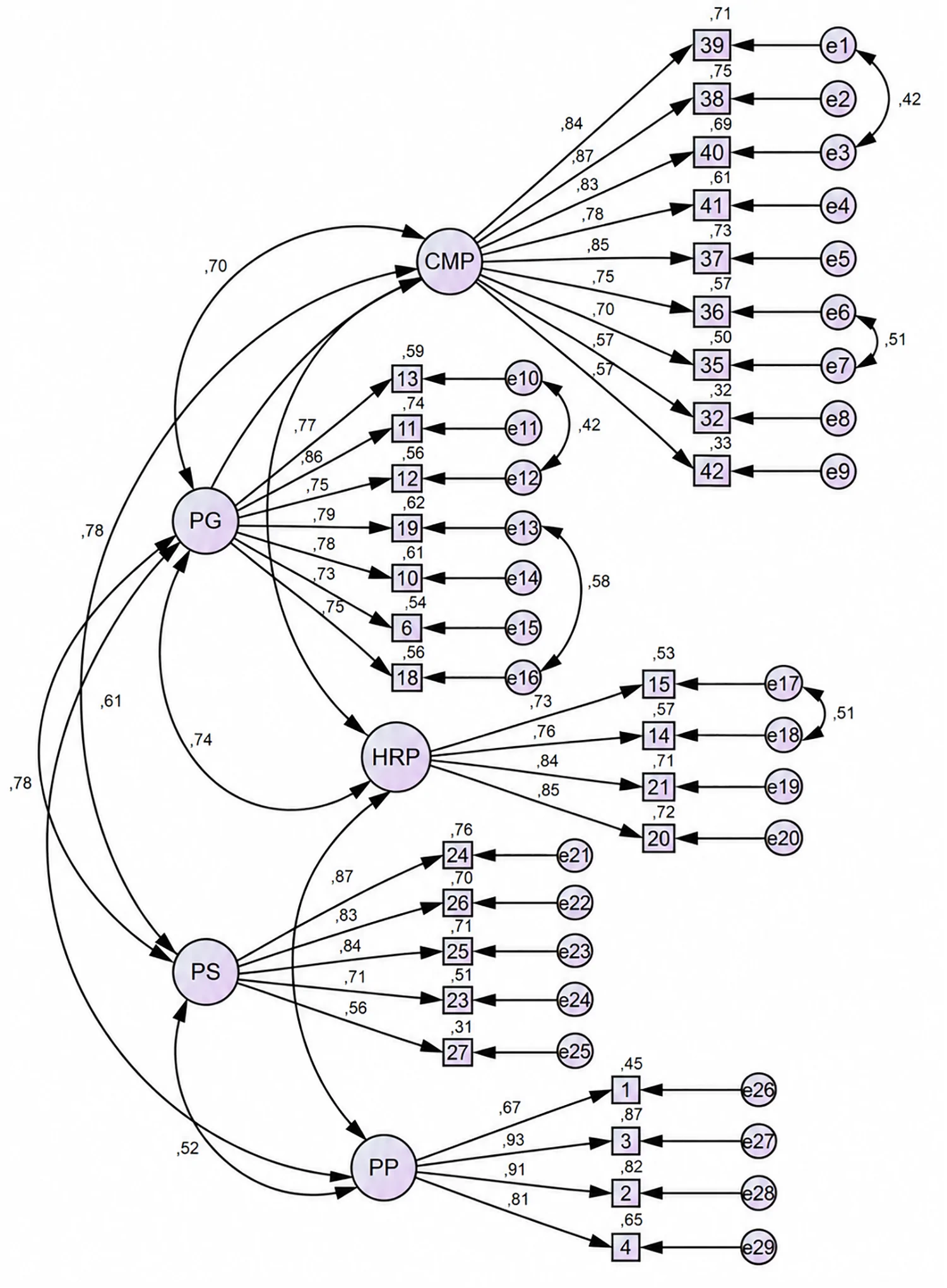
Global Climate Crisis Risk Perception Scale Eigenvalue Scree Plot.

The KMO value was sufficient resulting in 0.954. The chi-square value (chi-square:23972.069; df:990) obtained as a result of the Bartlett test was significant (p < 0.001). Based on the joint consideration of the scree plot, eigenvalues, cumulative variance explained, and conceptual interpretability, a five-factor solution was retained. All five retained factors had eigenvalues greater than 1 and together explained 69.40% of the total variance. The factor loadings of the items in the five-factor solution are presented in Table 3. The scree plot showed a gradual leveling off from the sixth component onward, which was consistent with the retention of five factors (Fig 1).

**Table 3:** Scale factor structure, exploratory variance values, eigenvalues, and Cronbach’s alpha values (n = 749).

Factors		Scale items	Factor loadings	Eigenvalue	Variance (%)	Cronbach’s αlpha
**Factor 1**	Crisis management perception	I39	.845	13.76	47.44	.919
		I38	.843			
		I40	.832			
		I41	.801			
		I37	.775			
		I36	.749			
		I35	.709			
		I32	.608			
		I42	.549			
**Factor 2**	Perceived gravity	I13	.764	2.24	7.73	.918
		I11	.739			
		I12	.665			
		I19	.598			
		I10	.577			
		I6	.551			
		I18	.528			
**Factor 3**	Health risk perception	I15	.799	1.66	5.74	.888
		I14	.782			
		I21	.717			
		I20	.685			
**Factor 4**	Perceived sensitivity	I24	.845	1.43	4,92	.858
		I26	.829			
		I25	.813			
		I23	.734			
		I27	.568			
**Factor 5**	Perceived probability	I1	.819	1.04	3.58	.883
		I3	.790			
		I2	.777			
		I4	.665			

Based on the contents of the items collected under the factors, appropriate nomenclature was bestowed on the factors. The five factors remerging as a result of EFA performed using the Direct oblimin and the factor loads collected within these items were shown in Table 3. The factor loads of the items were between 0.845 and 0.528. The factors were named appropriately based on the contents of the items. Factor 1, 9 items with loads ranging from 0.845 to 0.549 named as “Crisis management perception (CMP)”. Factor 2, 7 items with loads ranging from 0.764 to 0.528 named as “Perceived gravity (PG)”. Factor 3, 4 items with loads ranging from 0.799 and 0.685 named as “Health risk perception (HRP)”. Factor 4, 5 items with loads ranging from 0.845 and 0.568 named as “Perceived sensitivity (PS)”. Factor 5, 4 items with loads ranging from 0.819 and 0.665 named as “Perceived probability (PP)”.

First goodness-of-fit values resulting from CFA, CMIN/df = 6.272, AGFI = 0.775, CFI = 0.886, NFI = 0,900 and RMSEA = 0.084. To improve these values, covariances were formed according to the modification indices. Goodness-of-fit values obtained as a result of covariances are presented in Table 4.

**Table 4:** Goodness of fit criteria and the scale confirmatory factor analysis results.

Good fit indexes	Calculated value	Good fit criteria	Acceptable fit criteria	Degree of fit
**CMIN/df**	3.94	0 ≤ CMIN/df ≤ 2	2 < CMIN/df ≤ 5	Acceptable Fit
**RMSEA**	0.06	0 ≤ RMSEA≤0.05	0.05 < RMSEA≤0.08	Acceptable Fit
**AGFI**	0.85	0.90 ≤ AGFI≤1.00	0.85 ≤ AGFI<0.90	Acceptable Fit
**CFI**	0.94	0.97 ≤ CFI ≤ 1.00	0.90 ≤ CFI < 0.97	Acceptable Fit
**NFI**	0.92	0.95 ≤ CFI ≤ 1.00	0.90 ≤ CFI < 0.95	Acceptable Fit

**Fig 2:**
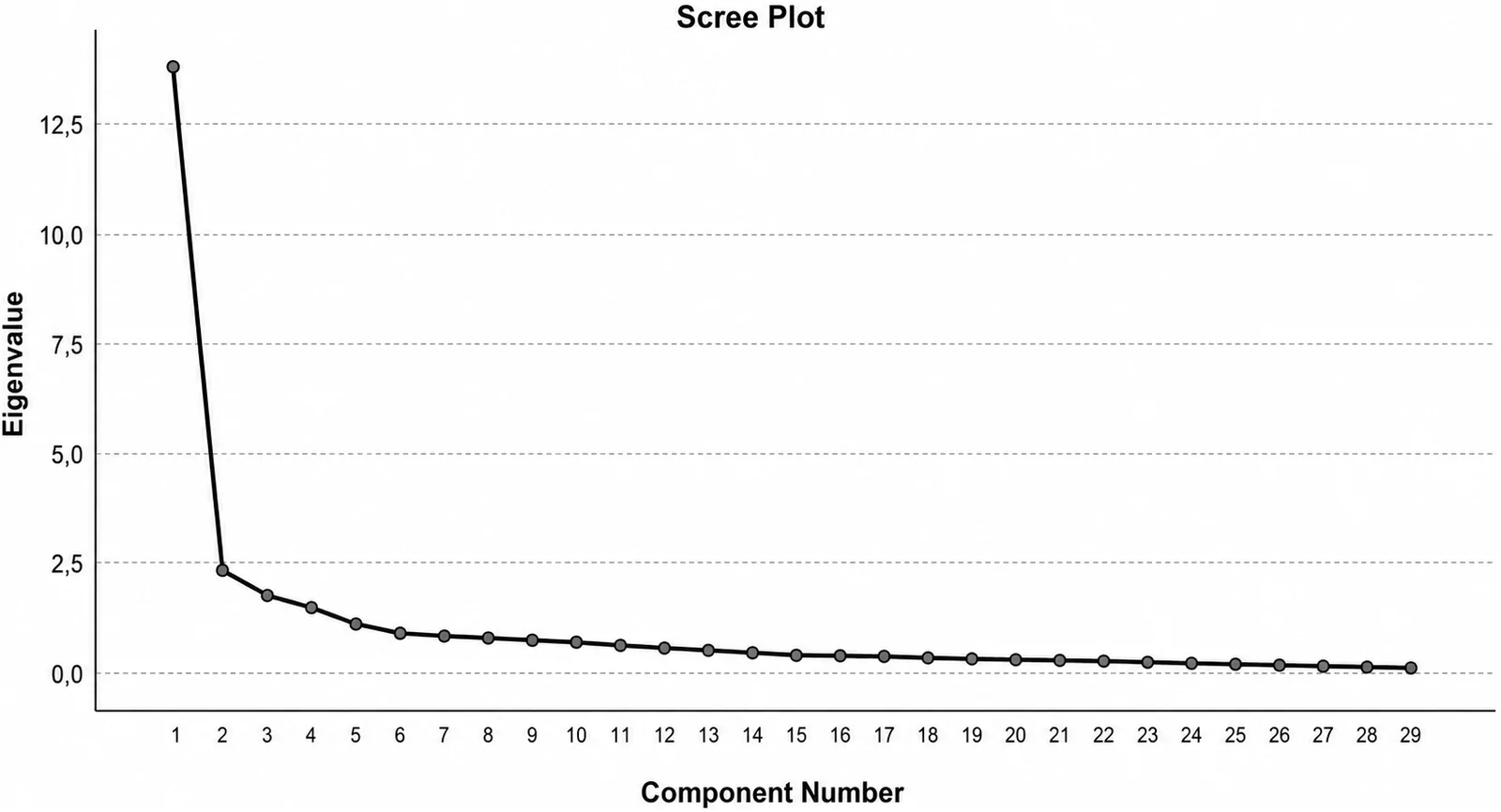
Confirmatory factor analysis results for the scale. (CMP) Crisis Management Perception; (PG) Perceived Gravity; (HRP) Health Risk Perception; (PS) Perceived Sensitivity; (PP) Perceived Probability).

The final version of the GCCRP-S model, standardize regression coefficients and squared multipl correlations values (R2) of the model are shown in Fig 2.

As shown in the diagram, the standardized regression coefficients are 0.570–0.868 for the CMP sub-dimension; 0.733–0.858 for PG; 0.727–0.851 for HRP; 0.558–0.869 for PS, and 0.669–0.935 for the PP sub-dimension. As a result of the analyses, it was seen that there were statistically significant and moderate correlations between the factors (coefficients, between 0.314 and 0.549; p < 0.001).

#### 3.2.4. Concurrent validity.

The correlation coefficients between GCCRP-S and other scales are shown in Table 5.

**Table 5:** Relationships Between the Global Climate Crisis Risk Perception Scale and the Other Scales.

Scales	Mean±SD	Median	Min-Max		1	2	3
**1. Global Climate Crisis Risk Perception Scale (GCCRP-S)**	228.3 ± 26.8	232	47-270	r p	1 .		
**2. Attitude Scale Towards Environmental Problems**	44.9 ± 6.5	45	23-55	r p	.579^**^ <0.001	1 .	
**3. Climate Change Awareness Scale**	33.4 ± 4.9	33	15-45	r p	.408^**^ <0.001	.328^**^ <0.001	1 .
**4. Climate Change Concern Scale**	31.3 ± 8.7	32	10-50	r p	.561^**^ <0.001	.447^**^ <0.001	.411^**^ <0.001

The table presents the Spearman correlation values between GCCRP-S and the other scales used in testing concurrent criterion validity. Moderate and statistically significant correlations were found between the candidate GCCRP-S and the other scales (p-values < 0.001). As the perception of global climate crisis risk increases, concern about climate change and positive attitudes towards environmental problems also increase. In addition, positive attitudes towards environmental problems rise with the level of awareness; individuals with higher awareness levels also tend to have higher levels of concern.

### 3.3. Reliability

#### 3.3.1. Internal Consistency.

The Cronbach’s α of the scale is 0.957. Values of the sub-dimensions are shown in **Table 3**. The item analysis values of the scale are shown in **Table 6**.

**Table 6:** Item Analysis Values of the Global Climate Crisis Risk Perception Scale (n = 749).

Item	Mean	SD	Skewness	Kurtosis	Corrected Item-Total Correlation	Cronbach’s α if Item Deleted
**I1**	5.31	.99	−1.91	4.21	.542	.957
**I2**	5.56	.72	−2.50	9.98	.701	.956
**I3**	5.54	.74	−2.59	10.73	.686	.956
**I4**	5.45	.79	−1.97	5.96	.678	.956
**I6**	5.57	.78	−2.74	10.38	.644	.956
**I10**	5.54	.79	−2.65	10.13	.699	.956
**I11**	5.50	.80	−2.35	7.93	.734	.955
**I12**	5.33	.95	−1.88	4.32	.679	.956
**I13**	5.40	.85	−2.02	5.79	.675	.956
**I14**	5.10	1.04	−1.39	2.20	.616	.956
**I15**	5.04	1.06	−1.39	2.27	.607	.956
**I18**	5.36	.84	−2.04	6.72	.714	.955
**I19**	5.42	.83	−2.14	6.75	.728	.955
**I20**	5.11	.97	−1.30	2.21	.687	.956
**I21**	5.00	1.04	−1.12	1.31	.670	.956
**I23**	5.19	1.01	−1.63	3.25	.594	.956
**I24**	5.37	0.86	−1.89	5.31	.658	.956
**I25**	5.39	0.89	−2.24	7.09	.648	.956
**I26**	5.36	0.87	−2.00	5.96	.641	.956
**I27**	4.71	1.18	−0.94	.76	.506	.958
**I32**	5.17	1.03	−1.51	2.35	.495	.957
**I35**	5.15	1.00	−1.49	2.88	.648	.956
**I36**	5.20	.91	−1.44	2.95	.690	.955
**I37**	5.42	.87	−2.13	6.33	.741	.955
**I38**	5.50	.80	−2.58	9.81	.723	.955
**I39**	5.51	.82	−2.52	8.80	.723	.955
**I40**	5.48	.82	−2.49	8.92	.720	.955
**I41**	5.47	.88	−2.52	8.26	.667	.956
**I42**	5.25	1.01	−1.72	3.52	.532	.957

#### 3.3.2. Stability.

The scale was applied again to 58 individuals who volunteered to participate in the retest, three weeks after the first application. ICC was determined as 0.940 (95% CI 0.898–0.964).

## 4. Discussion

This study developed and provided initial psychometric evidence for the GCCRP-S as a multidimensional instrument for assessing global climate crisis risk perception. The content-validity and pilot-testing stages supported the relevance, clarity, and internal consistency of the item pool and informed its refinement before the main application.The KMO value of 0.954 indicated excellent sampling adequacy and supported the suitability of the correlation matrix for factor analysis. Bartlett’s test of sphericity was also statistically significant (p < 0.001), indicating that the inter-item correlations were sufficient to proceed with factor analysis [58,94]. The combined evaluation of the scree plot, eigenvalues, cumulative variance explained, and conceptual interpretability supported a five-factor solution, which explained 69.40% of the total variance. The conceptually interpretable five-factor solution provided support for the construct validity of the GCCRP-S [95].

The CFA results indicated acceptable model fit across all reported goodness-of-fit indices, including CMIN/df (3.94), AGFI (0.85), CFI (0.94), NFI (0.92), and RMSEA (0.06) [88,89]. Considering the sample size of 749, the RMSEA value of 0.06 also supported an acceptable model fit [58,95]. Statistically significant and moderate correlations between the factors indicated that the dimensions were related but remained empirically distinguishable.The GCCRP-S demonstrated excellent internal consistency, with a Cronbach’s α coefficient of 0.957 for the overall scale and coefficients ranging from 0.858 to 0.919 for its sub-dimensions. The test-retest ICC of 0.940 also indicated strong temporal stability [58,66,92,93]. The moderate and statistically significant correlations between the GCCRP-S and measures of environmental attitudes, climate change awareness, and climate change concern indicated that these constructs were related but not redundant. Awareness primarily reflects the recognition and understanding of climate change, whereas concern predominantly represents an affective response. In contrast, the GCCRP-S conceptualizes climate crisis risk perception as a multidimensional construct integrating perceived probability and gravity, health-related risks, personal sensitivity, and perceptions of crisis management. The absence of high correlations therefore supports the interpretation that the GCCRP-S assesses a conceptually distinct but complementary construct. Although a five-factor conceptual framework was initially proposed during item development, the EFA was conducted without imposing this predefined structure. The resulting five-factor solution was interpreted and labeled according to the conceptual content of the items loading on each factor. The items that loaded under the first factor were relevant to implementing actions aimed at averting the global climate crisis by promoting cooperation at both individual and societal, public/private sector, country/government levels. These items also emphasized the urgent need to fight. Therefore, this factor was named “Crisis management perception (CMP)”. The items that load under the second factor were related to the extent of damage that the global climate crisis may cause on all people, plant and animal species, food, and water resources. Therefore, this factor was named “Perceived gravity (PG)”. The items that load under the third factor were related to the effects of the global climate crisis on human health, such as chronic diseases, infectious diseases, cancers, and premature deaths. Therefore, this factor was named “Health risk perception (HRP)”. The items that load under the fourth factor were related to individuals’ vulnerability, susceptibility, anxiety, and fear against the current or possible effects of the global climate crisis. Therefore, this factor was named “Perceived sensitivity (PS)”. The items that load under the fifth factor were related to individuals will be harmed or experience an adverse outcome as a result of exposure to current or potential effects of the global climate crisis. Therefore, this factor was named “Perceived probability (PP)”. The items, which were initially predicted to belong to the “Perceived probability”, “Perceived sensitivity” and “Crisis management perception” factors, were found to come together and create loads under the predicted factors as a result of EFA. The items that were initially predicted to belong to the “Perception of importance” factor were removed from the EFA because they did not meet the eligibility criteria, so this predicted factor was eliminated ().

EFA showed that the items that were predicted to belong to the “Perceived gravity” factor when creating the item pool, were divided into two factors. The four items that load together under a new factor were related to the effects of the GCC on human health. This showed that the participants perceived the GCC as a risk in terms of possible health effects. The emergence of Health Risk Perception as a distinct factor is theoretically consistent with health behavior theories such as the Health Belief Model and Protection Motivation Theory, in which perceived health consequences constitute a central determinant of risk appraisal and protective behavior. Rather than representing a post-hoc empirical artifact, this factor reflects participants’ differentiation of health-related threats from general environmental severity within their cognitive risk evaluation. The factor containing the remaining items was named “Perceived gravity” as initially predicted.

Risk perception constitutes a core element of several health behavior theories, including the Health Belief Model, Protection Motivation Theory, and Self-Regulation Theory. These frameworks suggest that individuals who perceive a situation as posing a significant threat are more likely to engage in preventive actions, adhere to recommended measures, and influence others to do the same. In a similar vein, within the context of the global climate crisis, individuals who perceive climate change as a serious and imminent threat are more motivated to adopt pro-environmental behaviors. These may include reducing carbon emissions, supporting climate policies, and participating in collective mitigation efforts. Higher levels of perceived climate risk are associated with greater behavioral engagement and compliance with environmental protection measures. Therefore, risk perception plays a pivotal role in shaping and motivating behavioral responses to climate change. Enhancing public awareness of climate-related risks can serve as a strategic lever to promote sustainable behavior at both individual and societal levels.

Existing measurement approaches have generally focused on specific aspects of climate-related perceptions, such as climate change awareness, worry or concern, or the cognitive, experiential, and sociocultural determinants of climate risk judgments [44,45,72,96–99]. Although these approaches provide valuable information about particular components of climate-related perceptions, they do not assess perceived probability, gravity, health risks, personal sensitivity, and crisis management perception together within a single multidimensional instrument. The GCCRP-S may therefore be preferred when the research objective is to obtain a broad profile of climate crisis risk perception across these five domains. In contrast, more focused instruments may be more appropriate when the primary aim is specifically to assess climate awareness, worry, concern, or another narrowly defined psychological response. The measurement of global climate crisis risk perception is essential for the adoption and support of international policies to combat the crisis. Studies have shown that people’s willingness to take protective actions is influenced by their perception of risk. Risk perception serves as a key factor in bridging the gap between national and international policies and individual and societal behavior, playing a crucial role in driving behavioral change [36].

Understanding how the global climate crisis is perceived may help policymakers and other stakeholders contextualize public responses to climate-related risks and better frame communication concerning national and international climate initiatives. In this respect, risk perception may serve as a relevant contextual factor for governmental and non-governmental organizations, the private sector, and educational institutions, particularly in awareness-building and risk-communication activities [36]. The GCCRP-S may provide a useful framework for understanding public perceptions of climate-related risks and may inform future policy-oriented research. As no behavioral outcomes or predictive validity analyses were included in the present study, any policy or behavioral implications of the GCCRP-S should be interpreted as prospective rather than evidence-based.

### 4.1. The superior features of the GCCRP-S development stages

A very large item pool, containing 64 items, was created; opinions were obtained from 22 specialists, experts in their fields, were obtained to determine the content validity; data were obtained through face to face interviews; the approach and positive attitudes of the individuals facilitated its performance; a higher number of participants than recommended in the literature was enrolled; a high degree of participation was achieved, the need for a scale measuring global climate crisis risk perception in society was confirmed, and the scale is acceptable to society; participants provided honest and correct answers to the questions/items in the data collection form, and there were no missing data; individuals from different segments of society in terms of education level and professions participated; the pilot study, the main application, and the test-retest procedure were all in accordance with all the criteria specified in the literature; the scale contains five sub-dimensions that can describe the risks in different areas associated with the global climate crisis; at least four items appear under each factor; the scale’s Cronbach α coefficient, EFA, CFA, and ICC values met the criteria required for a scale for scientific evaluation.

### 4.2. The limitations of the GCCRP-S development stages

Its single-center nature; stratification was performed only by gender and age; no stratification was carried out according to other sociodemographic characteristics (education level, occupation, income status). The GCCRP-S was developed in Turkish, but it can be translated into other languages and adapted to other societies with our permission. A translation of the scale was presented in the S1 File. We can provide insights in case of a cooperative study on this matter.

The study was conducted in a single district using convenience sampling, which limits population representativeness. In addition, individuals over the age of 60 and illiterate participants were excluded to ensure reliable comprehension of scale items during the initial development phase. While this approach strengthened internal validity, it may have reduced external validity by underrepresenting populations that may be particularly vulnerable to climate impacts. Therefore, further validation studies are warranted in older adults, low-literacy groups, and other vulnerable populations to extend the generalizability of the GCCRP-S.

From a methodological perspective, the use of Principal Component Analysis rather than common factor analysis and the application of Confirmatory Factor Analysis on the same dataset represent limitations of the present study. In addition, conducting multiple EFA iterations may increase the risk of capitalizing on chance, and the use of post-hoc error covariances in CFA should be interpreted with caution as they may reflect model overfitting. Accordingly, future research should employ split-sample or independent validation designs, utilize common factor analytic approaches, and conduct cross-validation to further strengthen the construct validity and generalizability of the GCCRP-S.

Reliability in the present study was primarily assessed using Cronbach’s alpha. To provide a more comprehensive evaluation of internal consistency and construct coverage, future validation studies are recommended to report additional reliability and validity indices, including McDonald’s omega, composite reliability, and average variance extracted (AVE).

## 5. Conclusion

The findings provide initial evidence supporting the validity and reliability of the GCCRP-S for assessing global climate crisis risk perception among adults aged 18–60 in the study population. This scale has 29 items and five sub-dimensions. Scores obtained from the scale are between 29–174. Higher scores indicate increased perceived risk related to the Global Climate Crisis.

The GCCRP-S may provide a useful framework for future research examining how individuals perceive different dimensions of climate-related risk. Its findings may also contribute contextual evidence to discussions on climate-risk communication and policy; however, its predictive and policy-related utility requires evaluation in independent and more diverse samples.

GCCRP-S can be used to evaluate the current perceptions of individuals and societies in studies on the global climate crisis. Future studies may examine the adaptation and validation of the GCCRP-S in different population groups, including policymakers and decision-makers. To convey the concepts more accurately, it can be applied to university students who might be the managers of the future. Today, it is seen that the global climate crisis disproportionately affects vulnerable groups. Therefore, it is anticipated that GCCRP-S will be guiding studies to be conducted in these groups.

## Supporting information

S1 File Global Climate Crisis Risk Perception Scale (GCCRP-S). This file contains the items of the Global Climate Crisis Risk Perception Scale (GCCRP-S).
(DOCX)
