# Supplementary material for: Development of the global climate crisis risk perception scale (GCCRP-S): A validity and reliability study
Source: PLoS One. 2026 Sep 9;21(9):e0321836. doi: 10.1371/journal.pone.0321836 (PMC13557399; doi:10.1371/journal.pone.0321836)
Supplement: Supplementary file 1 [file pone.0321836.s001.docx]

Global Climate Crisis Risk Perception Scale (GCCRP-S)

| **Global Climate Crisis Risk Perception Scale (GCCRP-S)** | **I Completely Agree** | **I Agree** | **I Partially Agree** | **I Partially Disagree** | **I Disagree** | **I Completely Disagree** |
| --- | --- | --- | --- | --- | --- | --- |
| **Perceived Probability** | | | | | | |
| 1. I am amazed by those who do not accept that the global climate crisis exists. |  |  |  |  |  |  |
| 2. The global climate crisis may harm social life. |  |  |  |  |  |  |
| 3. The global climate crisis may disrupt our quality of life. |  |  |  |  |  |  |
| 4. The global climate crisis may negatively affect my physical health. |  |  |  |  |  |  |
| **Perceived Gravity** | | | | | | |
| 5.The global climate crisis may negatively affect water resources. |  |  |  |  |  |  |
| 6. The global climate crisis concerns all people. |  |  |  |  |  |  |
| 7.Global climate crisis reduces the number of plant species. |  |  |  |  |  |  |
| 8.The global climate crisis causes the extinction of terrestrial animal species. |  |  |  |  |  |  |
| 9.The global climate crisis reduces the diversity of fish and other aquaculture species. |  |  |  |  |  |  |
| 10.The global climate crisis may cause a food crisis. |  |  |  |  |  |  |
| 11.The global climate crisis may cause a water crisis. |  |  |  |  |  |  |
| **Health Risk Perception** | | | | | | |
| 12.Global climate crisis causes infectious disease outbreaks. |  |  |  |  |  |  |
| 13. The global climate crisis makes it easier for me to catch infectious diseases. |  |  |  |  |  |  |
| 14. Chronic diseases and cancers may increase due to the global climate crisis. |  |  |  |  |  |  |
| 15. Premature deaths may increase due to the global climate crisis. |  |  |  |  |  |  |
| **Perceived Sensitivity** |  |  |  |  |  |  |
| 16. The change in seasonal characteristics makes me think. |  |  |  |  |  |  |
| 17. The decrease in woodland and forest areas worries me. |  |  |  |  |  |  |
| 18.I am worried about the increasing drought. |  |  |  |  |  |  |
| 19. The increasing air pollution in cities worries me. |  |  |  |  |  |  |
| 20. I think I will face the danger of hunger due to the global climate crisis. |  |  |  |  |  |  |
| 21. I support the work carried out against the global climate crisis. |  |  |  |  |  |  |
| **Crisis Management Perception** |  |  |  |  |  |  |
| 22.The global climate crisis should be a priority on the political agenda. |  |  |  |  |  |  |
| 23.The global climate crisis should be a priority on the public agenda. |  |  |  |  |  |  |
| 24.Urgent measures must be taken against the global climate crisis. |  |  |  |  |  |  |
| 25. Against the global climate crisis, the public and private sectors should attach importance to environmentally friendly technologies. |  |  |  |  |  |  |
| 26.Everyone should take responsibility against the global climate crisis. |  |  |  |  |  |  |
| 27.The public and private sectors should take responsibility together against the global climate crisis. |  |  |  |  |  |  |
| 28.All countries should cooperate against the global climate crisis. |  |  |  |  |  |  |
| 29.The concept of global climate crisis is not sufficiently understood by society. |  |  |  |  |  |  |
